# Effect of one-lung ventilation on end-tidal carbon dioxide during cardiopulmonary resuscitation in a pig model of cardiac arrest

**DOI:** 10.1371/journal.pone.0195826

**Published:** 2018-04-12

**Authors:** Dong Hyun Ryu, Yong Hun Jung, Kyung Woon Jeung, Byung Kook Lee, Young Won Jeong, Jong Geun Yun, Dong Hun Lee, Sung Min Lee, Tag Heo, Yong Il Min

**Affiliations:** 1 Department of Emergency Medicine, Chonnam National University Hospital, Gwangju, Republic of Korea; 2 Department of Emergency Medical Services, Honam University, Gwangju, Republic of Korea; Azienda Ospedaliero Universitaria Careggi, ITALY

## Abstract

Unrecognized endobronchial intubation frequently occurs after emergency intubation. However, no study has evaluated the effect of one-lung ventilation on end-tidal carbon dioxide (ETCO_2_) during cardiopulmonary resuscitation (CPR). We compared the hemodynamic parameters, blood gases, and ETCO_2_ during one-lung ventilation with those during conventional two-lung ventilation in a pig model of CPR, to determine the effect of the former on ETCO_2_. A randomized crossover study was conducted in 12 pigs intubated with double-lumen endobronchial tube to achieve lung separation. During CPR, the animals underwent three 5-min ventilation trials based on a randomized crossover design: left-lung, right-lung, or two-lung ventilation. Arterial blood gases were measured at the end of each ventilation trial. Ventilation was provided using the same tidal volume throughout the ventilation trials. Comparison using generalized linear mixed model revealed no significant group effects with respect to aortic pressure, coronary perfusion pressure, and carotid blood flow; however, significant group effect in terms of ETCO_2_ was found (*P* < 0.001). In the post hoc analyses, ETCO_2_ was lower during the right-lung ventilation than during the two-lung (*P* = 0.006) or left-lung ventilation (*P* < 0.001). However, no difference in ETCO_2_ was detected between the left-lung and two-lung ventilations. The partial pressure of arterial carbon dioxide (PaCO_2_), partial pressure of arterial oxygen (PaO_2_), and oxygen saturation (SaO_2_) differed among the three types of ventilation (*P* = 0.003, *P* = 0.001, and *P* = 0.001, respectively). The post hoc analyses revealed a higher PaCO_2_, lower PaO_2_, and lower SaO_2_ during right-lung ventilation than during two-lung or left-lung ventilation. However, the levels of these blood gases did not differ between the left-lung and two-lung ventilations. In a pig model of CPR, ETCO_2_ was significantly lower during right-lung ventilation than during two-lung ventilation. However, interestingly, ETCO_2_ during left-lung ventilation was comparable to that during two-lung ventilation.

## Introduction

Unrecognized endobronchial intubation frequently occurs following emergency intubation [1–3]. Multiple studies have suggested that clinical assessments, including chest auscultation, are unreliable in detecting endobronchial intubation [1,4,5]. Chest radiograph remains one of the definitive means to detect endobronchial intubation. However, this modality is not generally used during cardiopulmonary resuscitation (CPR) because obtaining chest radiograph inevitably requires chest compression interruption. Thus, inadvertent endobronchial intubation is likely to remain undetected during CPR.

End-tidal carbon dioxide (ETCO_2_) is a non-invasive measure of pulmonary perfusion during cardiac arrest. It has been shown to reflect changes in cardiac output during CPR and predict resuscitation outcomes, making it a useful guide for resuscitation efforts [6–12]. Currently, resuscitation guidelines recommend the use of physiological parameters, such as ETCO_2_, during CPR [13,14]. Under normal physiological conditions, alveolar ventilation is the principal determinant of alveolar carbon dioxide concentration in the lungs. The partial pressure of carbon dioxide within the alveoli is inversely related to the amount of alveolar ventilation. In the case of unrecognized endobronchial intubation, the total tidal volume is delivered to the intubated lung, resulting in doubling of ventilation/perfusion ratio in the intubated lung. Thus, theoretically, ventilation through an endotracheal tube inadvertently placed into the main stem bronchus would cause a decrease and an increase in alveolar carbon dioxide in the intubated and contralateral lungs, respectively. ETCO_2_ would decrease following endobronchial intubation because capnography measures the partial pressure of carbon dioxide only in the intubated lung. Several studies performed in a non-arrest animal model also reported a decrease in ETCO_2_ following one-lung ventilation achieved through endobronchial intubation or bronchial occlusion [15,16]. Meanwhile, the relationship between ETCO_2_ and alveolar ventilation becomes more complicated during CPR because chest compressions, as well as ventilations, influence the pulmonary gas exchange [17]. Thus, the effect of one-lung ventilation on ETCO_2_ during CPR may differ from that during the normal cardiac output state. However, to our knowledge, no study has evaluated the effect of one-lung ventilation on ETCO_2_ during CPR.

In this study, we compared the hemodynamic parameters, blood gases, and ETCO_2_ during one-lung ventilation with those during conventional two-lung ventilation in a pig model of CPR, to determine the effect of the former on ETCO_2_ during CPR. We hypothesized that ETCO_2_ would decrease during one-lung ventilation compared with two-lung ventilation.

## Materials and methods

This prospective, randomized, crossover study was conducted in 12 Yorkshire/Landrace cross pigs weighing 22.8 ± 3.2 kg, which were intubated with double-lumen endobronchial tube to achieve lung separation. Additionally, ETCO_2_ was assessed in one animal after tracheal and endobronchial intubations using a standard endotracheal tube. Thus, 13 pigs were used in this study. The Animal Care and Use Committee of Chonnam National University approved the protocol (CNU IACUC-H-2017-1). Animal care and experiments were conducted based on the author’s Institutional Animal Care and Use Committee Guidelines.

### Animal preparation

Following the administration of premedications (20 mg/kg ketamine and 2.2 mg/kg xylazine intramuscularly), the animals were placed in a supine position in a U-shaped trough and were orally intubated. Anesthesia was provided using 70%:30% N_2_O:O_2_ and 0.5–2% sevoflurane, which was titrated to prevent signs of pain (reactive wide pupils, tachycardia, and hypertension). A double-lumen catheter was inserted via the right femoral artery for blood pressure monitoring and blood sampling. The right external jugular vein was cannulated with an 8 F introducer sheath to monitor the right atrial (RA) pressure and insert a right ventricle (RV) pacing catheter. The right common carotid artery was surgically exposed, and an ultrasonic flow probe (Transonic Inc., Ithaca, NY, USA) was placed around it for carotid blood flow (CBF) measurement. An ETCO_2_ sample line (B40 Patient Monitor; GE Healthcare, Chalfont St Giles, UK) was connected to the ventilator circuit. In large pigs, the tip of the orally intubated tracheal tube does not reach the carina although the tube’s rostral end is positioned deep into the oral cavity. Furthermore, in our preliminary experience, placement of a tracheal tube into the desired main stem bronchus was extremely difficult during CPR because of the tracheal compression caused by chest compression and presence of oedema in the airways, which resulted in CPR interruptions for up to several minutes. For these reasons, a 35 F double-lumen endobronchial tube (Broncho-Cath^™^, Covidien, Mansfield, MA, USA) was placed under bronchoscopic guidance (Ambu^Ⓡ^ aScope^™^; Ambu A/S, Ballerup, Denmark) after tracheal tube removal, with the tip of the longer bronchial lumen placed in the left main stem bronchus while the tip of the shorter tracheal lumen remained in the trachea, to achieve lung separation during CPR. Immediately after the insertion, a fiberoptic bronchoscope was introduced into the tracheal lumen. While observing the carina, the tube position was adjusted until the bronchial cuff was just below the carina, and both the bronchial and tracheal cuffs were inflated to seal the left bronchus and trachea, respectively. We chose to place the bronchial lumen in the left main stem bronchus because the right upper lobe bronchus arose from the trachea immediately above the carina in our pigs. Throughout the rest of the preparation period, both lungs were ventilated using a Y-adapter for the proximal ends. After a 20 min stabilization period, baseline measurements were obtained, and vecuronium (0.05 mg/kg) was administered intravenously to inhibit the potential confounding effect of gasping.

### Experimental protocol

**Fig 1:**
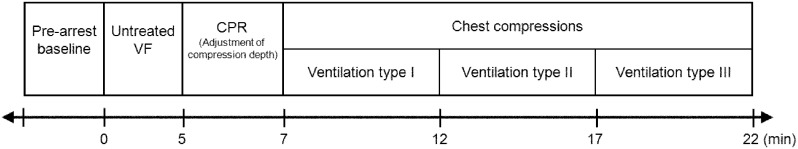
Experimental timeline. VF, ventricular fibrillation; CPR, cardiopulmonary resuscitation.

Ventricular fibrillation (VF) was induced by applying an electrical current (60 Hz, 30 mA alternating current) via an RV pacing catheter. After 5 min of untreated VF, the animals underwent a 2 min CPR period to wash out the accumulated carbon dioxide in the trachea while adjusting the chest compression depth. Chest compressions were delivered at a rate of 100 /min using a piston-driven chest compression device (Life-Stat; Michigan Instruments, Grand Rapids, MI, USA). The compression depth was adjusted to decrease the anterior–posterior diameter of the chest by 20%. Following the 2 min CPR period, the animals underwent three 5 min ventilation trials during CPR, with each animal receiving the following three types of ventilation based on a randomized crossover design: left-lung, right-lung, or two-lung ventilation (which refers to ventilation through the bronchial lumen, tracheal lumen, or both lumens using a Y-adapter, respectively) (Fig 1). The order of the ventilation types was counterbalanced and randomized using closed envelope method. Throughout this period, asynchronous positive-pressure ventilations with high-flow oxygen (14 l/min) were provided with a tidal volume of 10 ml/kg and rate of 10 /min using a volume-marked bag devised by Cho et al [18]. A tidal volume identical to that used for two-lung ventilation was utilized during right- and left-lung ventilations to simulate one-lung ventilation in unrecognized endobronchial intubation. During the right- or left-lung ventilation, the non-ventilated lumen was left open to the atmosphere. The investigator ventilating the animal was blinded to the ETCO_2_ level, but not to the ventilation type. The animals were not resuscitated after completion of the experiment, and thus an additional euthanasia procedure was not required in our study. Autopsy was routinely performed to ensure that each lung was adequately ventilated during one-lung ventilation.

### Measurements

The primary outcome was ETCO_2_ values, which were determined every 30 s by averaging the ETCO_2_ values for the preceding 30 s interval. Aortic and RA pressures were continuously monitored (CS/3 CCM; Datex-Ohmeda, Helsinki, Finland), and the data were transferred to a personal computer using S/5 Collect (Datex-Ohmeda, Helsinki, Finland). Coronary perfusion pressure (CPP) was calculated by subtracting the end-diastolic RA pressure from the simultaneous end-diastolic aortic pressure. Aortic systolic pressure, aortic diastolic pressure, and CPP were sampled at 30 s intervals by averaging pressures from five consecutive compressions. CBF was continuously monitored, and its value was sampled every 30 s. Arterial blood gases (Rapidlab 865; Bayer Health Care, Fernwald, Germany) were measured at the pre-arrest baseline and end of each ventilation type.

### Statistical analysis

Sample size was calculated based on the ETCO_2_ data (mean ± standard deviation [SD], 14.13 ± 5.70 mmHg; variance, 32.50) from a pilot study, where the ETCO_2_ values were 17.03 ± 2.77, 18.29 ± 3.12, and 7.08 ± 2.06 mmHg for two-lung, left-lung, and right-lung ventilations, respectively, and the calculated within-group variance was 25.17. We calculated that nine animals would be required to achieve a power of 80% at an ɑ of 0.05. Considering that each animal received all three interventions in a randomized counterbalanced order based on the study design, 12 animals were used for this study. Normally distributed variables were presented as mean ± SD, and a repeated-measure analysis of variance was performed. In contrast, non-normally distributed variables were presented as medians with interquartile ranges, and a Friedman test was conducted. Generalized linear mixed model was used to compare aortic and RA pressures, CPP, CBF, and ETCO_2_ during CPR. Pairwise comparison with Bonferroni adjustment was performed for post-hoc analysis. A *P* value of < 0.05 was considered significant.

### ETCO_2_ observation in one animal undergoing one- and two-lung ventilations using a standard endotracheal tube

The preparation and VF induction procedures were identical to those described above, except that tracheostomy was performed and a 6.5 mm internal diameter endotracheal tube (Hi-Lo; Mallinckrodt Medical, Athlone, Ireland) was placed into the tracheostomy stoma, instead of the double-lumen endobronchial tube. During 5 min of untreated VF, the endotracheal tube was advanced into the right main stem bronchus under bronchoscopic guidance. After 5 min of untreated VF, CPR was performed as described above, during which ventilation was provided through the endotracheal tube (right-lung ventilation). Subsequently, 5 min after the start of CPR, the endotracheal tube was pulled back until the cuff was just below the tracheostomy stoma so that both lungs were ventilated. Five minutes thereafter, the endotracheal tube was advanced into the left main stem bronchus while withholding CPR, and then chest compressions and ventilations (left-lung ventilation) were resumed. Five minutes thereafter, the endotracheal tube was pulled back again so that both lungs were ventilated. Five minutes thereafter, the endotracheal tube was advanced again into the right main stem bronchus while withholding CPR, and then chest compressions and ventilations (right-lung ventilation) were provided for 5 min.

## Results

**Fig 2:**
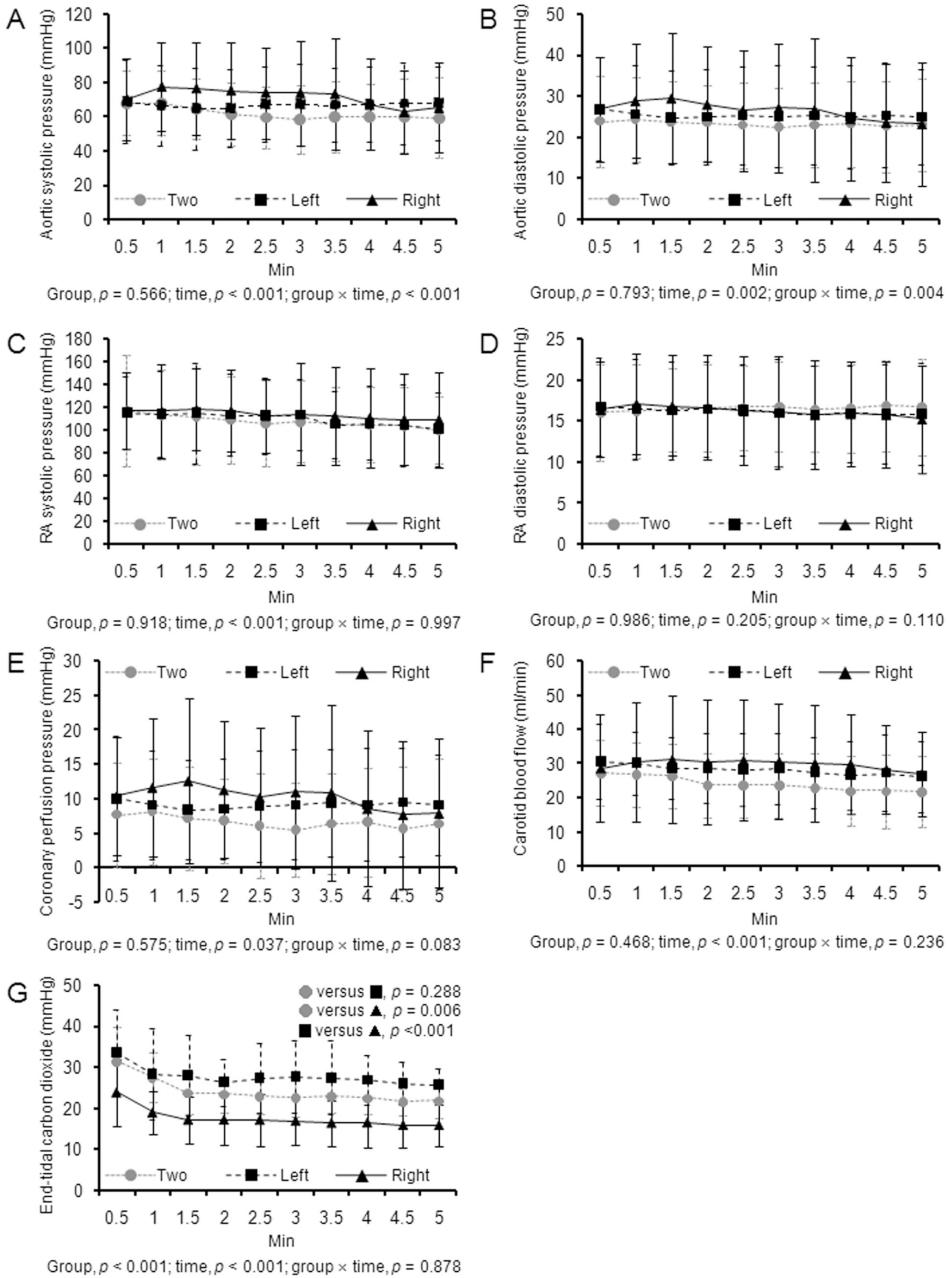
Hemodynamic parameters during two-lung, left-lung, and right-lung ventilations. (A) Aortic systolic pressure, (B) aortic diastolic pressure, (C) right atrial systolic pressure, (D) right atrial diastolic pressure, (E) coronary perfusion pressure, (F) carotid blood flow, (G) end-tidal carbon dioxide. RA, right atrial.

**Fig 3:**
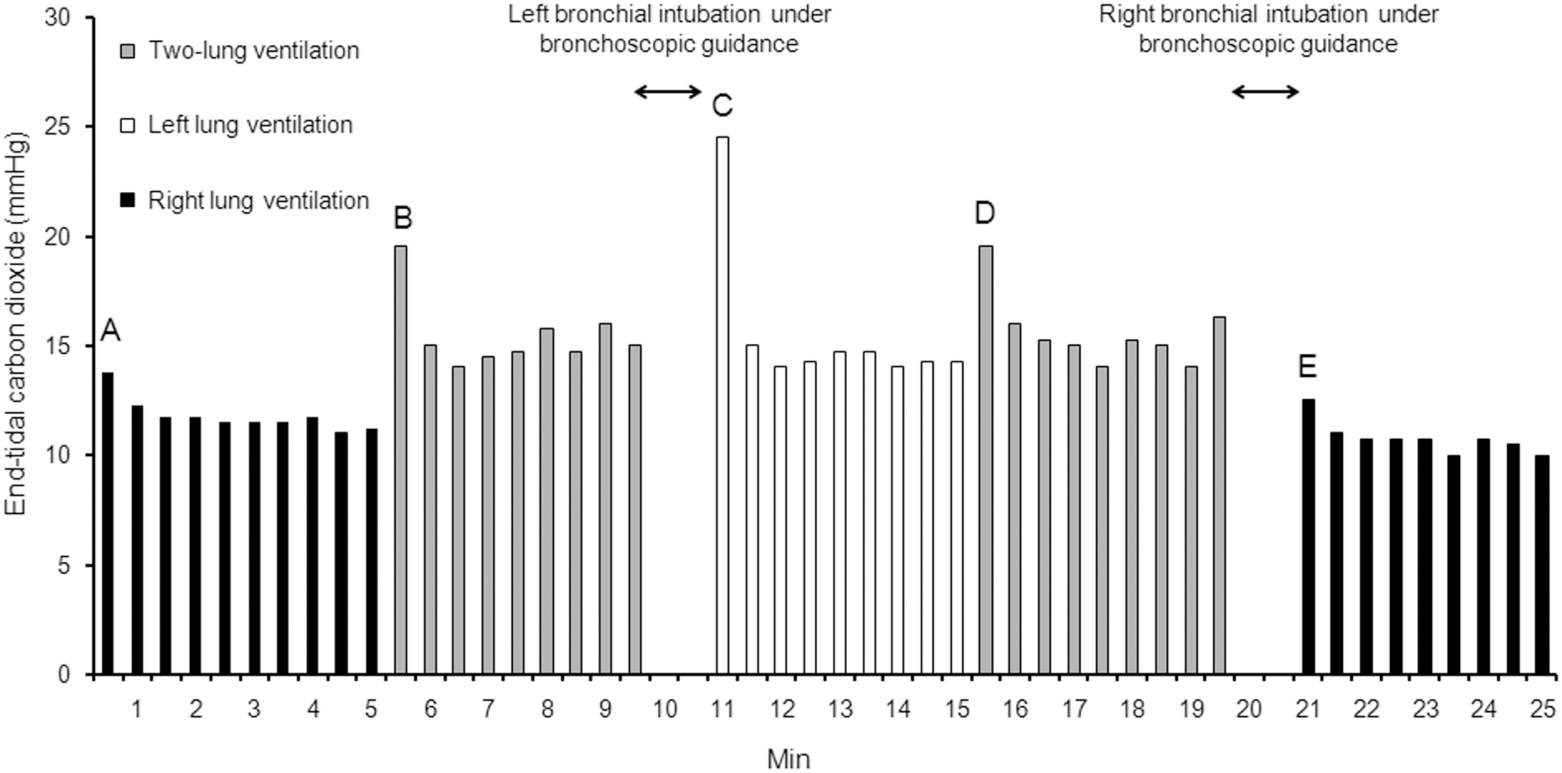
ETCO_2_ in an animal undergoing two-lung, left-lung, and right-lung ventilations via a standard endotracheal tube during cardiopulmonary resuscitation. At the onset of right-lung ventilation (point A), ETCO_2_ was 13.8 mmHg, which stabilized within 1 min and remained between 11.0 and 11.8 mmHg during the rest of the right-lung ventilation. Immediately after the endotracheal tube was pulled back to the tracheal position (point B), ETCO_2_ increased to 19.5 mmHg and remained between 14.0 and 16.0 mmHg after stabilization. At the onset of left lung ventilation (point C), ETCO_2_ was 24.5 mmHg and remained between 14.0 and 15.0 mmHg after stabilization. Immediately after the endotracheal tube was pulled back to the tracheal position (point D), ETCO_2_ increased to 19.5 mmHg and remained between 14.0 and 16.3 mmHg after stabilization. Immediately after the endotracheal tube was advanced into the right main bronchus (point E), ETCO_2_ decreased to 12.5 mmHg and remained between 10.0 and 11.0 mmHg after stabilization.

Table 1 shows the pre-arrest baseline measurements. Fig 2 displays the aortic and RA pressures, CPP, CBF, and ETCO_2_ during each ventilation type. Comparison using generalized linear mixed model revealed no significant group effects with respect to aortic and RA pressures, CPP, and CBF; however, significant group effect in terms of ETCO_2_ was noted (*P* < 0.001). In the post hoc analyses, a significant difference in ETCO_2_ was found between the right-lung and two-lung ventilations (*P* = 0.006) and between the left-lung and right-lung ventilations (*P* < 0.001). However, no difference in ETCO_2_ was noted between the left-lung and two-lung ventilations (*P* = 0.288). Table 2 shows the arterial blood gases obtained at the end of each ventilation type. A significant difference in the partial pressure of arterial carbon dioxide (PaCO_2_), partial pressure of arterial oxygen (PaO_2_), and oxygen saturation (SaO_2_) was observed between the three types of ventilation (*P* = 0.003, *P* = 0.001, and *P* = 0.001, respectively). The post hoc analyses revealed a higher PaCO_2_, lower PaO_2_, and lower SaO_2_ during right-lung ventilation than during two-lung or left-lung ventilation. However, the post hoc analyses also showed that the levels of these blood gases did not differ between the left-lung and two-lung ventilations. A significant difference in the PaCO_2_-ETCO_2_ gradient was observed between the three types of ventilation (*P* = 0.020). The post hoc analyses showed a higher PaCO_2_-ETCO_2_ gradient during right-lung ventilation than during two-lung or left-lung ventilation. However, the PaCO_2_-ETCO_2_ gradient did not differ between the left-lung and two-lung ventilations. Fig 3 displays the ETCO_2_ values of one animal undergoing two-lung, left-lung, and right-lung ventilations via a standard endotracheal tube during CPR.

**Table 1:** Pre-arrest baseline measurements.

Aortic systolic pressure (mmHg)	129.0 (113.8–136.0)
Aortic diastolic pressure (mmHg)	86.1 ± 13.8
Right atrial systolic pressure (mmHg)	11.5 (9.5–14.3)
Right atrial diastolic pressure (mmHg)	7.0 (4.5–9.8)
Heart rate (/min)	98.1 ± 15.0
pH	7.450 ± 0.061
PaCO_2_ (mmHg)	42.1 ± 6.0
PaO_2_ (mmHg)	102.3 ± 28.0
HCO_3_^−^ (mmol/l)	28.6 ± 2.9
SaO_2_ (%)	97.5 (93.1–98.8)
End-tidal carbon dioxide (mmHg)	36.0 ± 3.7
Carotid blood flow (ml/min)	262.3 ± 77.4

**Table 2:** Arterial blood gases and PaCO_2_-ETCO_2_ gradient obtained at the end of each ventilation type.

	Two-lung ventilation (n = 12)	Left-lung ventilation (n = 12)	Right-lung ventilation (n = 12)	*P*
pH	7.162 ± 0.118	7.157 ± 0.117	7.146 ± 0.084	0.749
PaCO_2_ (mmHg)	64.7 ± 14.1^a^	64.8 ± 12.5^a^	73.6 ± 14.0^b^	0.003
PaO_2_ (mmHg)	59.5 (53.1–75.8)^a^	70.0 (55.9–92.5)^a^	41.5 (38.7–48.1)^b^	0.001
HCO_3_^−^ (mmol/l)	22.4 ± 4.3	22.2 ± 3.9	24.5 ± 3.8	0.087
SaO_2_ (%)	76.0 (65.6–85.1)^a^	81.3 (60.0–93.6)^a^	42.3 (39.3–55.0)^b^	0.001
PaCO_2_-ETCO_2_ gradient	42.8 ± 12.4^a^	39.0 ± 12.8^a^	57.8 ± 12.4^b^	0.020

Data are presented as mean ± standard deviation or medians with interquartile ranges.

Superscripts a and b represent pairwise post hoc analyses. For a particular variable, values with different superscripts are significantly different, whereas those with common superscripts are not significantly different from each other.

## Discussion

The present study evaluated the effect of one-lung ventilation occurring in the case of unrecognized endobronchial intubation in a pig model of CPR, and the results showed that ETCO_2_ and PaO_2_ were significantly lower but PaCO_2_ was significantly higher during right-lung ventilation than during two-lung ventilation. However, interestingly, ETCO_2_ and arterial blood gas values during left-lung ventilation were comparable to those during two-lung ventilation. A consistent finding was also observed when one-lung and two-lung ventilations were simulated using a standard endotracheal tube.

A previous study performed in a non-arrest animal model reported that one-lung ventilation resulted in a significant but transient decrease in ETCO_2_ [16]. Johnson et al. conducted a study to assess the changes in ETCO_2_ after left main stem bronchus occlusion in open-chested dogs, and their findings revealed that ETCO_2_ decreased at the onset of bronchial occlusion but returned to its pre-occlusion values within 3 min as hypoxic pulmonary vasoconstriction (HPV) redistributed blood flow from the non-ventilated to the ventilated lung [16]. In the present study, right-lung ventilation resulted in a significant decrease in ETCO_2_ compared with the two-lung ventilation. This decrease appeared to remain relatively constant throughout the 5 min ventilation trial. We speculate that the persistent decrease in ETCO_2_ in our study could be attributed to inhibition of HPV during CPR with high-flow oxygen [19].

We initially expected that left-lung ventilation would result in decreases in ETCO_2_ that are equivalent to those observed in the right-lung ventilation. However, in contrast to our expectation, ETCO_2_ during left-lung ventilation was significantly higher than that during right-lung ventilation and did not differ from that during two-lung ventilation. In fact, ETCO_2_ was higher in left-lung ventilation than in two-lung ventilation, although the difference did not reach statistical significance. ETCO_2_ is determined by total body carbon dioxide production, alveolar ventilation, and pulmonary perfusion. The total body carbon dioxide production was unlikely to differ between the right-lung and left-lung ventilations. Thus, the observed difference in ETCO_2_ between the right-lung and left-lung ventilations has two possible explanations: different alveolar ventilation or different pulmonary perfusion. Although ventilations were delivered with the same tidal volume and respiratory rate, the actual alveolar ventilation could have differed between the right-lung and left-lung ventilations. In the present study, the gradient between PaCO_2_ and ETCO_2_, which is regarded as an index of dead space ventilation (although it is also affected by pulmonary perfusion), was significantly higher in right-lung ventilation than in left-lung ventilation. Thus, the difference in ETCO_2_ between the right-lung and left-lung ventilations might be attributable to the difference in the amount of dead space ventilation. The volume of anatomic and apparatus dead space might have differed between the right-lung and left-lung ventilations because of the structural difference of the bronchial and tracheal lumens of a double-lumen tube. However, consistent results regarding the ETCO_2_ during right-lung and left-lung ventilations were observed in the animal that underwent ventilation trials using a standard endotracheal tube, thus making this possibility less likely. Another, more likely explanation is that left-lung ventilation might have resulted in less atelectasis and more effective alveolar recruitment, consequently improving ventilation in the relatively better-perfused, dependent part of the lung. Studies suggest that a substantial amount of atelectasis occurs in the dependent part of the lung during CPR [20,21]. Because the left lung is smaller than the right lung, the ventilation volume relative to lung volume would be larger in left-lung ventilation than in right-lung ventilation, despite the same tidal volume delivery. Thus, the relatively higher left-lung ventilation volume might have opened the dependent, atelectatic part of the lung, which receives more perfusion than the non-dependent ventral part of the lung owing to gravity, thereby improving ventilation/perfusion matching in the dependent part of the lung.

On the other hand, the difference in ETCO_2_ between the right-lung and left-lung ventilations might be attributable to different pulmonary perfusion. However, the total pulmonary blood flow was unlikely to differ between the right- and left-lung ventilations. Chest compression depth remained constant among the three ventilation types. Although cardiac output was not measured in the present study, aortic pressure, CPP, and CBF did not differ among the three ventilation types. The observed difference in ETCO_2_ between the left-lung and right-lung ventilations may be explained by the unequal distribution of pulmonary blood flow between the right and left lungs. In normal subjects, the left lung is slightly less perfused than the right lung, as the left lung is smaller than the right lung [22]. However, the left pulmonary artery of pigs, similar to that of humans, represents a direct continuation of the pulmonary trunk and the main pulmonary artery is directed towards the left pulmonary artery as shown in S1 Movie. Thus, the leftward direction of the main pulmonary artery itself may favor flow toward the left lung. Moreover, the left pulmonary artery courses posteriorly, while the right pulmonary artery follows a longer and more horizontal course as it crosses the mediastinum. Thus, our finding can be explained by supposing that, in addition to the effect of the leftward direction of the main pulmonary artery, the hydrostatic pressure due to gravity also causes the blood to be carried preferentially into the left pulmonary artery, and also supposing that the effect of these factors is significantly operational in low-pulmonary blood flow states such as during CPR, while this effect is negligible in normal blood flow states. However, our explanations remain speculative at best. Thus, our finding should be considered as hypothesis-generating.

Our findings may have potential clinical implications. First, the changes in ETCO_2_ caused by endobronchial intubation may confound the interpretation of ETCO_2_ during CPR. In particular, the decrease in ETCO_2_ following right endobronchial intubation may lead to inappropriate resuscitative effort termination. Second, the results of our study also suggest the importance of verifying correct endotracheal tube position in preventing hypoxemia during CPR. During one-lung anesthesia in otherwise normal subjects, adequate oxygenation is achieved despite one-lung ventilation due to a high inspired oxygen concentration. However, in the present study, PaO_2_ fell to a dangerous level (41.5 mmHg [38.7–48.1]) following right-lung ventilation. This finding indicates that inadvertent right endobronchial intubation can have a catastrophic effect on oxygenation during CPR despite the use of 100% oxygen because pulmonary gas exchange is already impaired during CPR [23]. To date, the optimal PaO_2_ level during CPR remains to be determined. However, a study that investigated the association between PaO_2_ during CPR and survival to hospital admission among 145 adult cardiac arrest patients reported that hypoxemia, defined as PaO_2_ ≤ 60 mmHg, was associated with decreased rate of survival to hospital admission [24].

Our study has several limitations. First, this study was conducted on pigs. Thus, the results should be verified in humans. Second, in the present study, care was taken to ensure that the endobronchial tube did not abut against the bronchial wall and the same tidal volume was delivered throughout the ventilation trials. However, endobronchial intubation in clinical settings can result in varying ventilation patterns depending on the tube tip position. If an endotracheal tube is inserted too deep into the main bronchus, the tube itself can cause obstruction of a lobar bronchus. In contrast, the non-intubated lung can be partially ventilated through the Murphy’s eye in cases when only the tip of endotracheal tube is positioned in the main stem bronchus. Third, the non-ventilated lumen was left open to the atmosphere during the one-lung ventilation trials, which might have facilitated the collapse of the non-ventilated lung. This might also have facilitated passive ventilation of the non-ventilated lung induced by chest compressions. Thus, our one-lung ventilation trials might not have precisely replicated the clinical setting of endobronchial intubation. Fourth, a randomized crossover design was utilized to reduce the number of animals used for this study. Previous studies suggested that changes in the non-ventilated lung, such as atelectasis, persisted even after restoration of ventilation [16,25]. Thus, the effects of one-lung ventilation might have persisted even after the completion of one-lung ventilation trial, which might have affected the results. Fifth, one-lung ventilation using a double-lumen endobronchial tube might exert different effects from that using a standard endotracheal tube. However, adequate respiratory excursions of the lungs were found during ventilation through the double-lumen tube at autopsy. Sixth, we could not determine the effect of one-lung ventilation on resuscitability.

## Conclusions

In conclusion, ETCO_2_ was significantly lower during right-lung ventilation than during two-lung ventilation in a pig model of CPR. However, interestingly, the ETCO_2_ level during left-lung ventilation was comparable to that during two-lung ventilation. Further studies are required to confirm our findings and elucidate the underlying mechanism.

## Supporting information

S1 Data Raw data. (XLSX)

S1 File ARRIVE guidelines checklist. (DOC)

S1 Movie Computed tomography (CT) showing the course of the pulmonary artery in a pig undergoing cardiopulmonary resuscitation (CPR). CT was performed immediately after withholding CPR. Note the contrast material preferentially filling the left pulmonary artery.
(GIF)
