# Supplementary figures and images for: Effect of one-lung ventilation on end-tidal carbon dioxide during cardiopulmonary resuscitation in a pig model of cardiac arrest
Source: PLoS One. 2018 Apr 12;13(4):e0195826. doi: 10.1371/journal.pone.0195826 (PMC5897021; doi:10.1371/journal.pone.0195826)

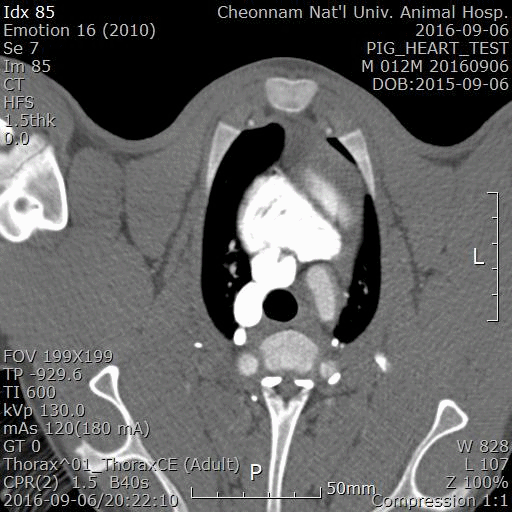

Supplement: S1 Movie — CT was performed immediately after withholding CPR. Note the contrast material preferentially filling the left pulmonary artery. (GIF) [file pone.0195826.s003.gif]
